# Causal role of immune cells in generalized anxiety disorder: Mendelian randomization study

**DOI:** 10.3389/fimmu.2023.1338083

**Published:** 2024-01-09

**Authors:** Zhen Ma, Min Zhao, Huanghong Zhao, Nan Qu

**Affiliations:** ^1^ Department of Neurology, The First Affiliated Hospital of Henan University of Traditional Chinese Medicine, Zhengzhou, China; ^2^ Department of Neurology, Henan Provincial Hospital of Traditional Chinese Medicine, Zhengzhou, China

**Keywords:** generalized anxiety disorder, immunity, causal inference, MR analysis, sensitivity

## Abstract

**Background:**

Generalized anxiety disorder (GAD) is a prevalent emotional disorder that has received relatively little attention regarding its immunological basis. Recent years have seen the widespread use of high-density genetic markers such as SNPs or CNVs for genotyping, as well as the advancement of genome-wide association studies (GWAS) technologies, which have facilitated the understanding of immunological mechanisms underlying several major psychiatric disorders. Despite these advancements, the immunological basis of GAD remains poorly understood. In light of this, we aimed to explore the causal relationship between immune cells and the disease through a Mendelian randomization study.

**Methods:**

The summary information for GAD (Ncase=4,666, Ncontrol=337,577) was obtained from the FinnGen dataset. Summary statistics for the characterization of 731 immune cells, including morphological parameters (MP=32), median fluorescence intensity (MFI=389), absolute cells (AC=118), and relative cells (RC=192), were derived from the GWAS catalog. The study involved both forward MR analysis, with immune cell traits as the exposure and GAD as the outcome, and reverse MR analysis, with GAD as the exposure and immune cell traits as the outcome. We performed extensive sensitivity analyses to confirm the robustness, heterogeneity, and potential multi-biological effects of the study results. Also, to control for false positive results during multiple hypothesis testing, we adopted a false discovery rate (FDR) to control for statistical bias due to multiple comparisons.

**Results:**

After FDR correction, GAD had no statistically significant effect on immunophenotypes. Several phenotypes with unadjusted low P-values are worth mentioning, including decreased PB/PC levels on B cells(β=-0.289, 95%CI=0.044~0.194, *P*=0.002), reduced PB/PC AC in GAD patients (β=-0.270, 95% CI=0.77~0.92, *P*=0.000), and diminished PB/PC on lymphocytes (β=-0.315, 95% CI=0.77~0.93, *P*=0.001). GAD also exerted a causal effect on CD27 on IgD-CD38br (β=-0.155,95%CI=0.78~0.94,*P*=0.002), CD20-%B cell (β= -0.105,95% CI=0.77~0.94, *P*=0.002), IgD-CD38br%lymphocyte(β=-0.305, 95%CI=0.79~0.95, *P*=0.002), FSC-A level on granulocytes (β=0.200, 95%CI=0.75~0.91, *P*=8.35×10^−5^), and CD4RA on TD CD4+(β=-0.150, 95% CI=0.82~1.02, *P*=0.099). Furthermore, Two lymphocyte subsets were identified to be significantly associated with GAD risk: CD24+ CD27+ B cell (OR=1.066,95%CI=1.04~1.10,*P*=1.237×10^−5^),CD28+CD4+T cell (OR=0.927, 95%CI=0.89~0.96, *P*=8.085×10^−5^).

**Conclusion:**

The study has shown the close association between immune cells and GAD through genetic methods, thereby offering direction for future clinical research.

## Introduction

Generalized Anxiety Disorder (GAD) is a pervasive mental health issue characterized by persistent, uncontrollable worry about events or activities (1, 2). Typically emerging in childhood or adolescence, GAD tends to persist if left untreated (3). It is often linked with other mental illnesses, such as depression, resulting in social, occupational, and educational challenges, increased healthcare utilization, and a heightened risk of substance abuse (4, 5). Consequently, it significantly impacts an individual’s overall health and daily functioning.

Current research has revealed the intricate relationship between anxiety disorders and the immune system. Laboratory findings have shown that abnormal secretion of inflammatory markers such as TNF-α and interleukin-6 (IL-6) can lead to elevated anxiety levels (6). Additionally, the activation and functional changes of immune cells, such as monocytes and T cells, can impact the neurotransmitter system, thereby influencing emotions and anxiety states (7). These studies suggest significant potential in exploring immune regulation for controlling anxiety disorders (8). However, for individuals with GAD, it remains a worthwhile area of research to investigate whether the persistent presence of anxiety mood and state is influenced by deeper genetic variations. Some scholars propose the involvement of acquired genetic and epigenetic risks in explaining the differential susceptibility to anxiety disorders, considering their highly complex and polygenic nature (9). However, only a few risk loci for anxiety disorders have been identified thus far. Furthermore, studies have found evidence of familial heritability in GAD and have focused on candidate genes such as 5-HTT, 5-HT1A, MAOA, and BDNF to explore the clinical genetics of GAD (10). Research also aims to address the high non-response or partial response of GAD patients to existing pharmacological treatments by seeking safer and more effective approaches through pharmacogenetics (PGx) (11). Genome-wide association studies (GWAS) play a crucial role in analyzing genetic variations across the entire genome in large cohorts, identifying expected genetic loci and pathways, and enhancing our understanding of the complex genetic factors underlying diseases (12). This will also contribute to the in-depth exploration of the relationship between susceptibility, immune inflammation, and genetic background in GAD patients in our study.

Mendelian randomization (MR) is a statistical mean primarily used to infer epidemiological causality based on Mendelian genetic principles (13). In the Mendelian randomization method, it is crucial to ensure the logical order of causality (14). Previous observational research have revealed certain links between immune cell traits and anxiety disorders, supporting the hypothesis of their association (15, 16). The study conducted a thorough two-sample MR analysis to explore the causal relationship between immune cell traits and GAD.

## Materials and methods

### Study design

**Figure 1:**
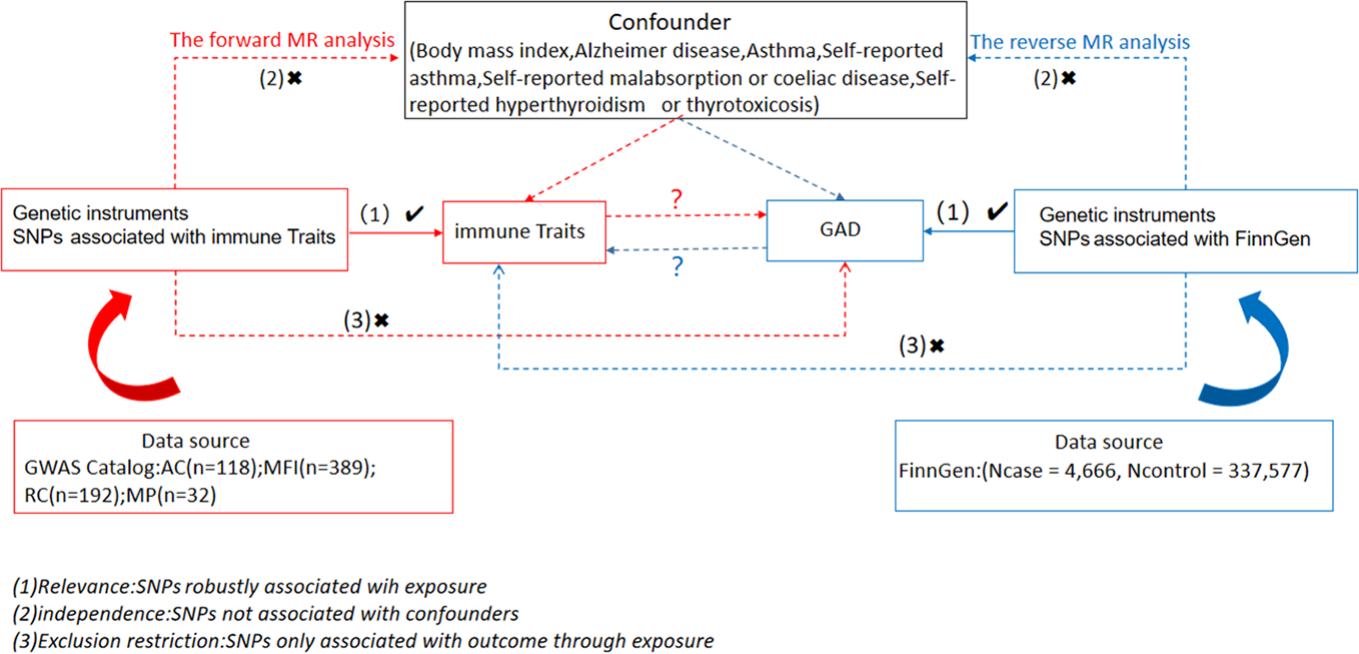
Overview of this bidirectional MR study design.

We examined causal associations between 731 immune cell traits and GAD using two-sample MR analyses. MR utilizes genetic variation as a surrogate for risk varies and requires validated instrumental variables (IVs) to satisfy three key assumptions in causal inference (1): Exposure is directly linked to genetic variation (2); there is no genetic link between exposure and outcome that is a potential confounder and (3) there is no genetic influence on outcome through channels unrelated to exposure (**Figure 1**). Our study was authorized by the appropriate institutional review board and informed permission was obtained from participants.

### Sources of immunity-spanning GWAS data

Summary statistics for all immunologic characteristics in the GWAS catalog (from accession numbers GCST0001391 to GCST0002121) are readily available (17). GWAS involves 3,757 nonoverlapping European individuals. A high-density array based on a reference panel of Sardinian sequences (18) was estimated for approximately 22 million SNPs and tested for correlation after controlling for covariates such as age, age^2, and sex. A number of 731 immunophenotypes were examined, including relative cell counts (RC) (192), morphologic parameters (MP) (32), absolute cell counts (AC) (118), and median fluorescence intensities (MFI) representing surface antigen levels (389). Of these, MP features included CDC and TBNK panels, whereas MFI, RC, and AC features included B cells, CDC, T cell maturation stage, myeloid cells, monocytes, and TBNK (T cells, B cells, natural killer proteins).

### Data sources from the genome-wide association study for GAD

The summary of GWAS statistics for GAD was obtained from FinnGen (https://www.finngen.fi/en). This summary was derived from a study involving 55,293 European individuals (Females=36,531, Males=18,494) with a median age (years) at first onset of GAD of 37.54 (Females=36.46, Males=40.62). The study encompassed a total of 342,243 samples (Ncase=4,666, Ncontrol=337,577), and the GWAS analysis incorporated over 500,000 phenotypic data points associated with GAD, identifying more than 16 million independent single nucleotide polymorphisms (SNPs).

### Selection of instrumental variables

A selection of instrumental variables (IVs) (version v1.90) (chain disequilibrium [LD] r2 threshold < 0.1 at a distance of 500 kb) (19) was used to modify these SNPs. 1000 Genomes Project was used as a reference panel for the calculation of the LD r2. 5 × 10-8 is the new GAD significance threshold. To assess IV strength and mitigate weak instrumental biases. The F statistic was calculated. The length of the independent variable (IV) for immunophenotypes ranged from 3 to 1,643 and explained on average 0.137% (0.009~0. 995%) of the variance in the relevant immune characteristics.

### Statistical analysis

The R version 4.3.1 program (http://www.Rproject.org) was used in all studies. To specifically assess the causal relationship between the 731 immunophenotypes and GAD, we performed median-based weighted analyses (20), pattern-based weighted analyses (21), and inverse variance weighted analyses (IVW) (22) using the “Mendelian Randomization” software (version 0.4.3) (23). Instrumental heterogeneity between variables was assessed using Cochran’s Q statistic and its p-value (IV) and combined with the MR-Egger method for horizontal multidimensionality, which was recognized if the intercept term was large (24). Meanwhile, in the MR-PRESSO package, we used the technique of robust MR multidirectional entropy residuals and outliers (MR-PRESSO) (25) to find and remove horizontal multidirectional entropy outliers that may seriously affect the estimation results. After removing these SNPs, we ran the IVW analysis again. In addition, we looked for SNPs with suggestive associations (*P*<10^-5^) with these risk factors on the Phenoscanner V2 Web site (http://www.phenoscanner.medschl.cam.ac.uk/). Lastly, we used funnel plots and scatter plots. Scatter plots showed that outliers had minimal effect on the data, whereas funnel plots showed a high degree of association and a lack of heterogeneity.

## Results

### Examination of the causal relation of GAD onset on immunophenotypes

**Figure 2:**
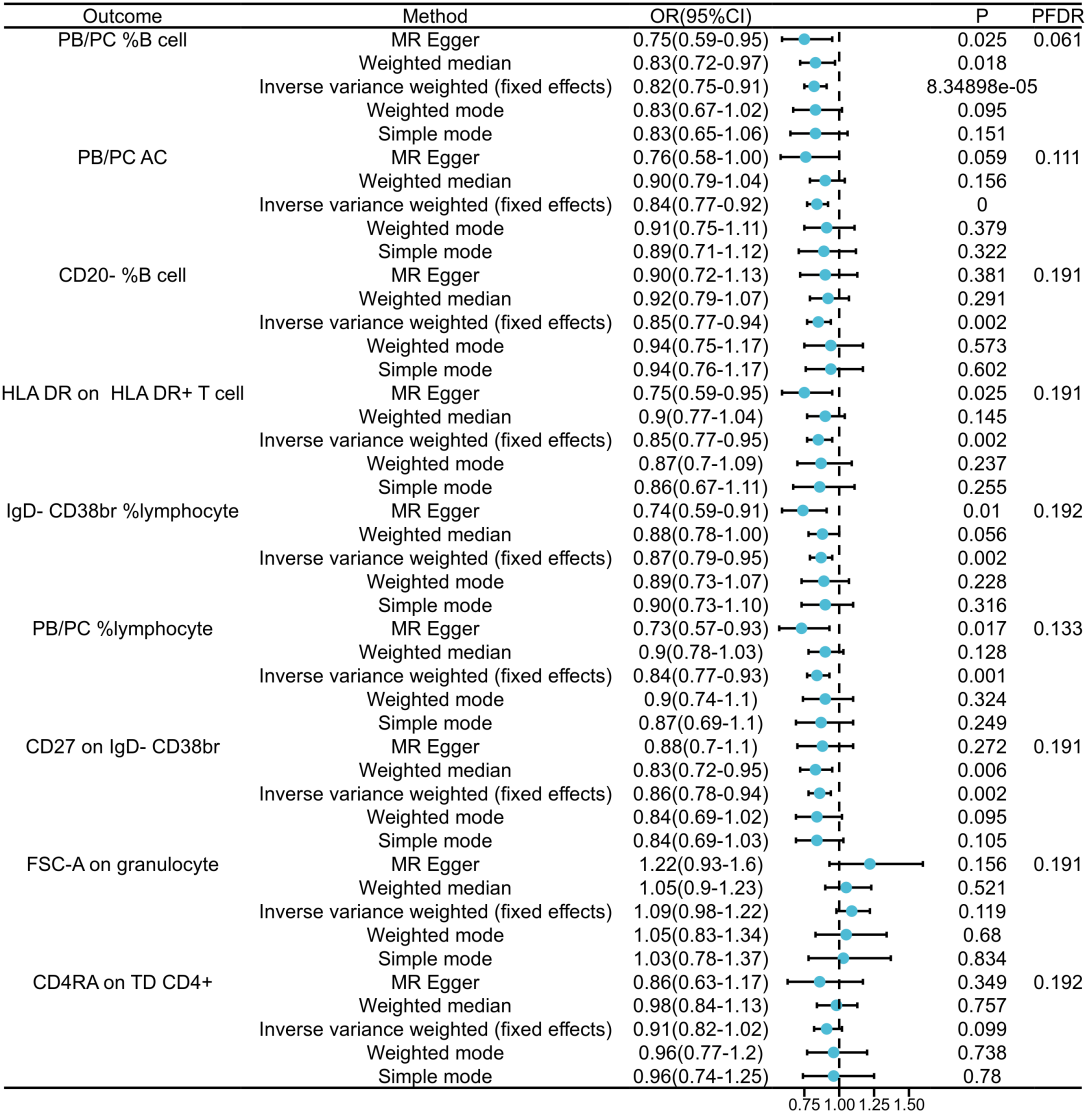
Forest plots showed the causal relations between GAD and immune cell traits.

In our exploration of GAD’s causal effects on immunophenotypes, we utilized the IVW method as the principal analysis in a two-sample MR analysis. Despite conducting multiple test adjustments via the FDR method, we did not identify any immune traits at a significance level of 0.05.Nonetheless, we detected nine suggestive immunophenotypes at a significance level of 0.20: two in the B cell panel, two in the lymphocyte panel, two in the CD38br panel, one in the PB/PC AC panel, one in the monocyte panel, and one in the T cell panel. Our findings suggest that GAD pathogenesis may decrease PB/PC levels in B cells (β=-0.289, 95% CI=0.044~0.194, *P*_FDR_=0.061, *P*=0.002, **Figure 2**, **Supplementary Tables 1**, **2**). as well as decreased PB/PC AC levels (β=-0.270, 95% CI=0.77~0.92, *P*_FDR_=0.111, *P*=0.000, **Figure 2**, **Supplementary Tables 1**, **2**).PB/PC of lymphocytes was also decreased in GAD patients (β=-0.315, 95% CI=0.77~0.93, *P*_FDR_=0.133, *P*= 0.001, **Figure 2**, and **Supplementary Tables 1** **2**). the causal effect of GAD on CD20- %B cells on transitional cells was estimated to be 0.85 (β= -0.105, 95% CI=0.77~0.94, *P*_FDR_=0.191, *P*=0.002, **Figure 2**, and **Supplementary Tables 1**, **2**). A similar association was found on CD27 of IgD-CD38br (β=-0.155, 95% CI=0.78~0.94, *P*_FDR_=0.191, *P*=0.002, **Supplementary Table 2**), and a positive association was also observed on IgD-CD38br% lymphocytes (β=-0.305, 95% CI=0.79~0.95, *P*_FDR_=0.192, *P*=0.002, **Supplementary Table 2**). Among the immune cell types positively associated with GAD, our findings suggest that the occurrence of GAD may increase the levels of FSC-A in granulocytes (β=0.200, 95% CI=0.75~0.91, *P*_FDR_=0.191, *P*=8.35×10-5, **Figure 2** and **Supplementary Tables 1**, **2**) and CD4RA in TD CD4+ (β=- 0.150, 95% CI=0.82~1.02, *P*_FDR_=0.192, *P*=0.099, **Supplementary Table 2**). The results of the other three methods and sensitivity analyses confirmed the robustness of the observed causal associations (**Supplementary Tables 1**, **3**). Specifically, the MR-Egger intercept and MR-PRESSO global tests ruled out the possibility of horizontal pleiotropy (**Supplementary Table 1**). In addition, scatterplots and funnel plots showed the stability of the results (**Supplementary Figure 2**).

### Examination of the causal relation of immunophenotypes on GAD

**Figure 3:**
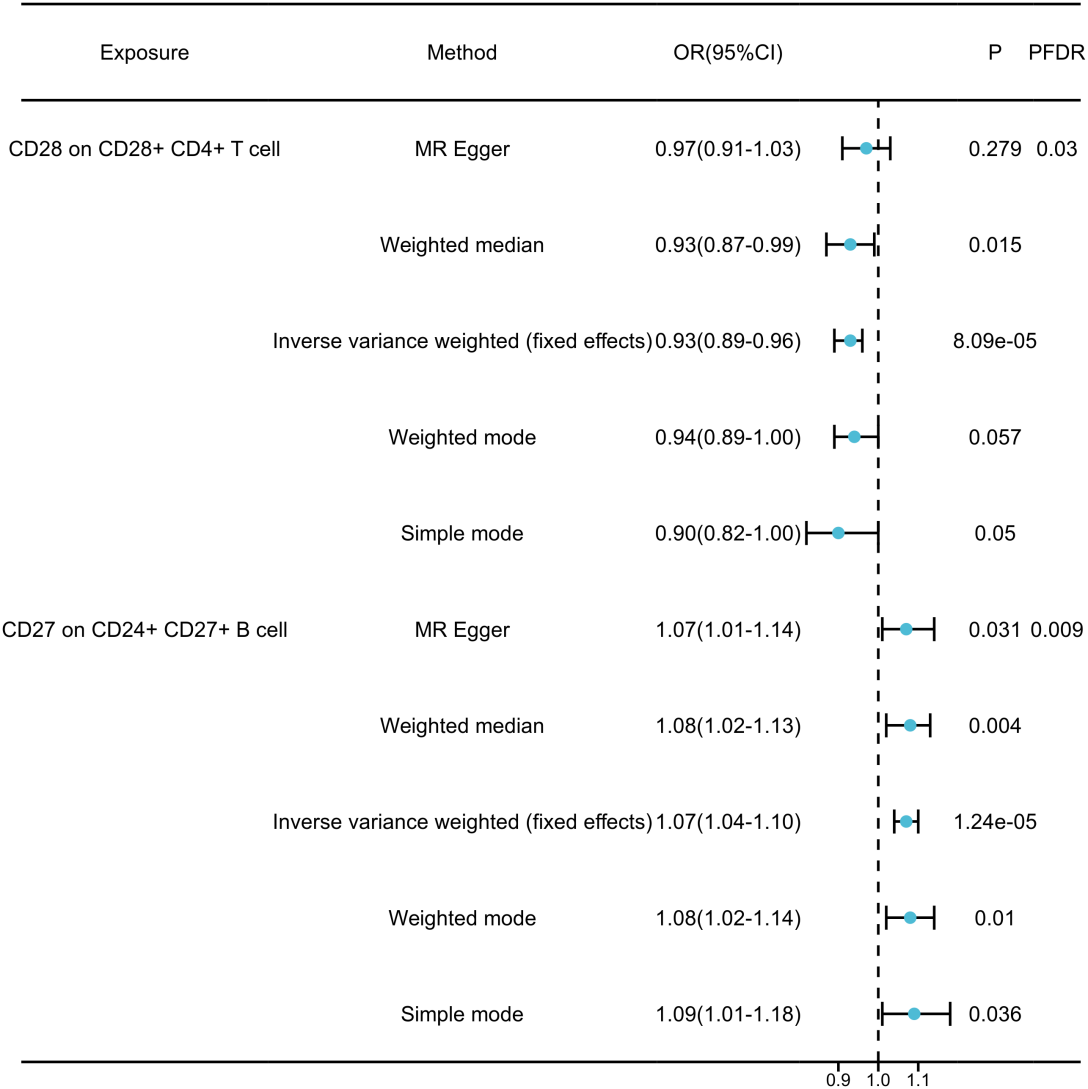
Forest plots showed the causal associations between immune cell traits and GAD by using different methods.

By FDR correction (*P*_FDR_<0.05), we found two immunophenotypes to be protective against generalized anxiety disorder: CD24+CD27+B cells and CD28+CD4+T cells. In particular, the ratio of CD24+CD27+B cells to GAD risk (OR) was assessed with the IVW method and was 0.94 (95% CI=1.04-1.10,*P*_FDR_=0.009, *P*=1.237×10-5, **Supplementary Table 4**). Three other methods yielded similar results: weighted mode (OR=1.077, 95% CI=1.02~1.14, *P*=0.010); weighted median (OR=1.075, 95% CI=1.02~1.13, *P*=0.004); and MR-Egger (OR=1.070, 95% CI=1.01~1.15, *P* =0.031). By applying the IVW technique, the OR of CD28+CD4+ T cells on GAD risk was calculated to be 0.064 (95% CI=0.89~0.96, *P*_FDR_=0.030, *P*=8.085×10-5, **Supplementary Table 4**). The results were similar for weighted mode (OR=0.944, 95% CI=0.89~1.00, *P*=0.057), weighted median (OR=0.927, 95% CI=0.87~0.99, *P*=0.015), and MR-Egger (OR=0.965, 95% CI=0.91~1.03, *P*=0.279). In addition, for all four associations, the MR-Egger intercept and MR-PRESSO global tests excluded the notion of horizontally collapsed products. Sensitivity analyses provided comprehensive details that validated the strength of the causal relationships found (**Figure 3**, **Supplementary Table 5**). The stability of the data was further demonstrated using scatterplots and funnel plots (**Supplementary Figure 1**).

## Discussion

We investigated the causal relationship between 731 immune cell characteristics and GAD using a large amount of publicly available genetic data. To date, this is the only MR investigation that has examined the causal connection between multiple immune phenotypes and GAD. The study included four immune trait categories (MFI, RC, AC, and MP). Among them. GAD showed a causal effect on nine immunophenotypes (*P*_FDR_< 0.20) and two immunophenotypes showed a strong causal effect on GAD (*P*_FDR_ < 0.05).

Our research indicates that an increased PB/PC%B cell percentage is associated with a reduced risk of GAD. PB/PC refers to two types of cells: plasmablasts (precursors of plasma cells) and plasma cells, and PB/PC%B cells represent the percentage of B cells in peripheral blood and spleen. Under normal circumstances, the ratio of PB/PC to lymphocytes in peripheral blood remains at a relatively high level, which is crucial for antibody production and immune cell clearance (26). Fluctuations in PB/PC%B cells often impact immune regulation and the intensity of immune responses (27), for instance, clinically predicting an increased risk of developing multiple sclerosis due to excessive immune reactions when the baseline PB/PC percentage falls below 0.1% (28), another study on anxiety comorbidity in adolescents showed a significant increase in lymphocyte ratios, suggesting a potential link between anxiety disorders and inflammatory responses (29).

The link between HLA-DR (human leukocyte antigen-DR) and neuropsychiatric disorders has attracted considerable attention. HLA-DR gene variants have been associated with susceptibility to specific neuropsychiatric disorders, such as schizophrenia and autism spectrum disorders (30). According to earlier research, HLA-DR may be a significant factor in the immunological abnormalities connected to GAD (31). The abnormally elevated expression of peripheral blood CD3~+ HLA-DR cells in patients with anxiety disorders and its correlation with immune disorders in patients suggests that it may have an impact on the development of GAD through its involvement in the modulation of immune responses.

Distinguishing specific cell subgroups is particularly important for immune profiling, such as the use of CD27-expressing IgD-CD38br cells to differentiate various lymphocyte subgroups. Studies have indicated the involvement of HLA-G5 as an immune regulatory molecule in the inflammatory cell response of anxiety disorders (32). The close association between the exposure to HLA-G5 and the decreased expression of IgD-CD38br cell subgroups highlights the potential role of immune cell subgroups in mediating anxiety disorders.

Reduced CD28 on CD28+ CD4+ T cells is linked to the usage of GAD as an exposure factor. The majority of research studies have demonstrated that patients with GAD have higher than normal blood levels of inflammatory cytokines, that the degree of clinical anxiety symptoms is significantly and positively correlated with the patient’s age (33), peripheral blood levels of IL-2, IL-4, and TF-alpha, and that CD28+ CD4+ T cells inhibit persistent inflammatory processes, thereby reducing the negative symptoms associated with GAD (34). Conversely, the percentage of CD24+ CD27+ B cells was significantly higher in GAD patients compared to healthy controls. This could be attributed to B cell activating factor (35), which regulates B cell survival and differentiation and plays a regulatory role in both natural and adaptive immunity. It is also involved in the development of a number of chronic inflammatory disorders, so it is likely that CD24+ CD27+ B cells also influence the inflammatory process of GAD via this pathway.

This study employed two-sample Mendelian randomization analysis using data from a large genomic research cohort of approximately 342,243 individuals, assuring great statistical efficiency. The outcomes of the study were based on genetic instrumental variables, and causal inferences were conducted by various robust Mendelian randomization analysis techniques, which were unaffected by horizontal pleiotropy and other variables. Moreover, to control for false positive results during multiple hypothesis testing, we adopted a false discovery rate (FDR) to control for statistical bias due to multiple comparisons.

However, This study does have several drawbacks, though. First, a thorough evaluation of horizontal pleiotropy is still difficult to achieve, even after several sensitivity studies. Second, stratified population analyses were not feasible due to the lack of individual-level data. Third, the study’s reliance on European databases limits the generalizability of the findings to other ethnic groups. Finally, The study’s flexible outcome assessment criteria may have led to more false positives, but they also made it easier to evaluate the full extent of the strong relationship between immunological traits and GAD. Overall, a randomized controlled trial of GAD would be the next step in this study in order to reduce the potential impact of confounding factors and thus obtain a higher level of evidence for causality.

## Conclusion

In summary, our comprehensive bidirectional MR analysis has revealed causal links between various immunophenotypes and GAD, shedding light on the intricate web of relationships between GAD and the immune system. Moreover, Reverse causality, other variables, and other unavoidable confounding factors have all been successfully reduced in impact by our study, offering a fresh perspective for researchers to delve into the biological underpinnings of GAD and potentially pave the way for early intervention and treatment strategies. These findings expand the realm of psychoimmunology and offer valuable insights for GAD prevention.

## Data availability statement

The original contributions presented in the study are included in the article/**Supplementary Material**. Further inquiries can be directed to the corresponding authors.

## Author contributions

MZ: Data curation, Writing – original draft. ZM: Formal Analysis, Writing – original draft. HZ: Methodology, Writing – original draft. NQ: Validation, Writing – review & editing.
