# Supplementary figures and images for: Causal role of immune cells in generalized anxiety disorder: Mendelian randomization study
Source: Front Immunol. 2024 Jan 9;14:1338083. doi: 10.3389/fimmu.2023.1338083 (PMC10803460; doi:10.3389/fimmu.2023.1338083)

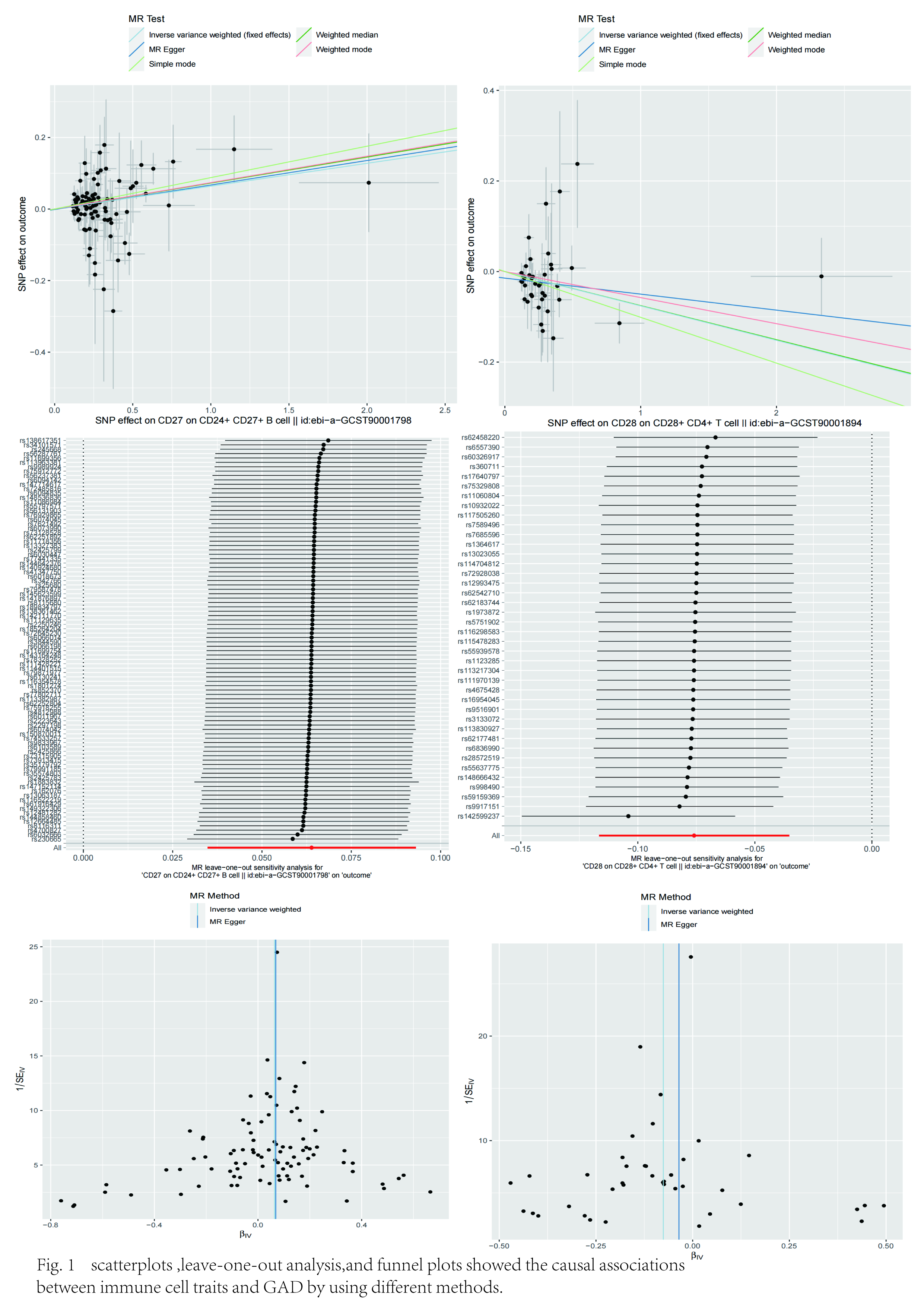

Supplement: Supplementary file 1 [file Image_1.tif]

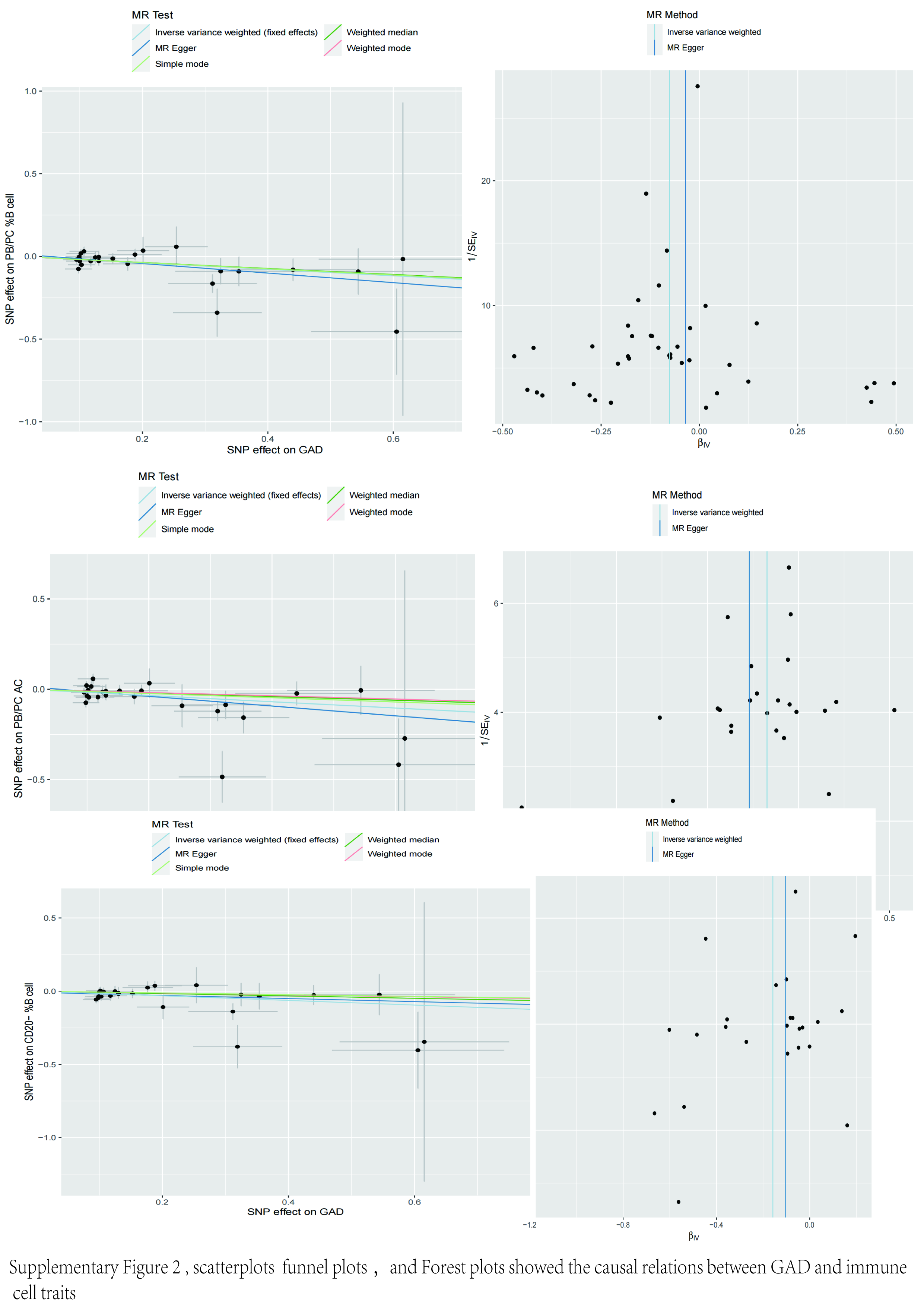

Supplement: Supplementary file 2 [file Image_2.tif]

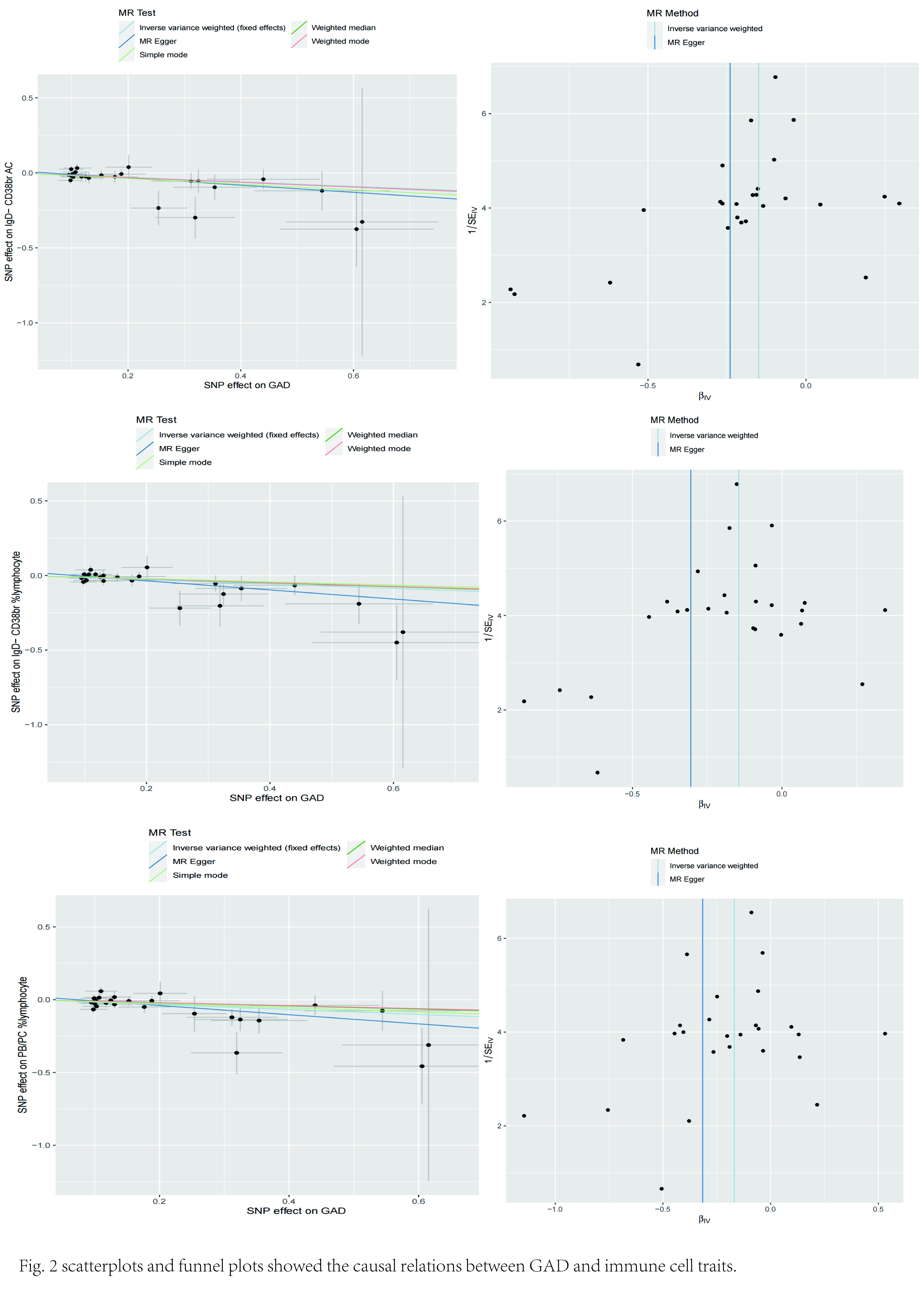

Supplement: Supplementary file 3 [file Image_3.tif]

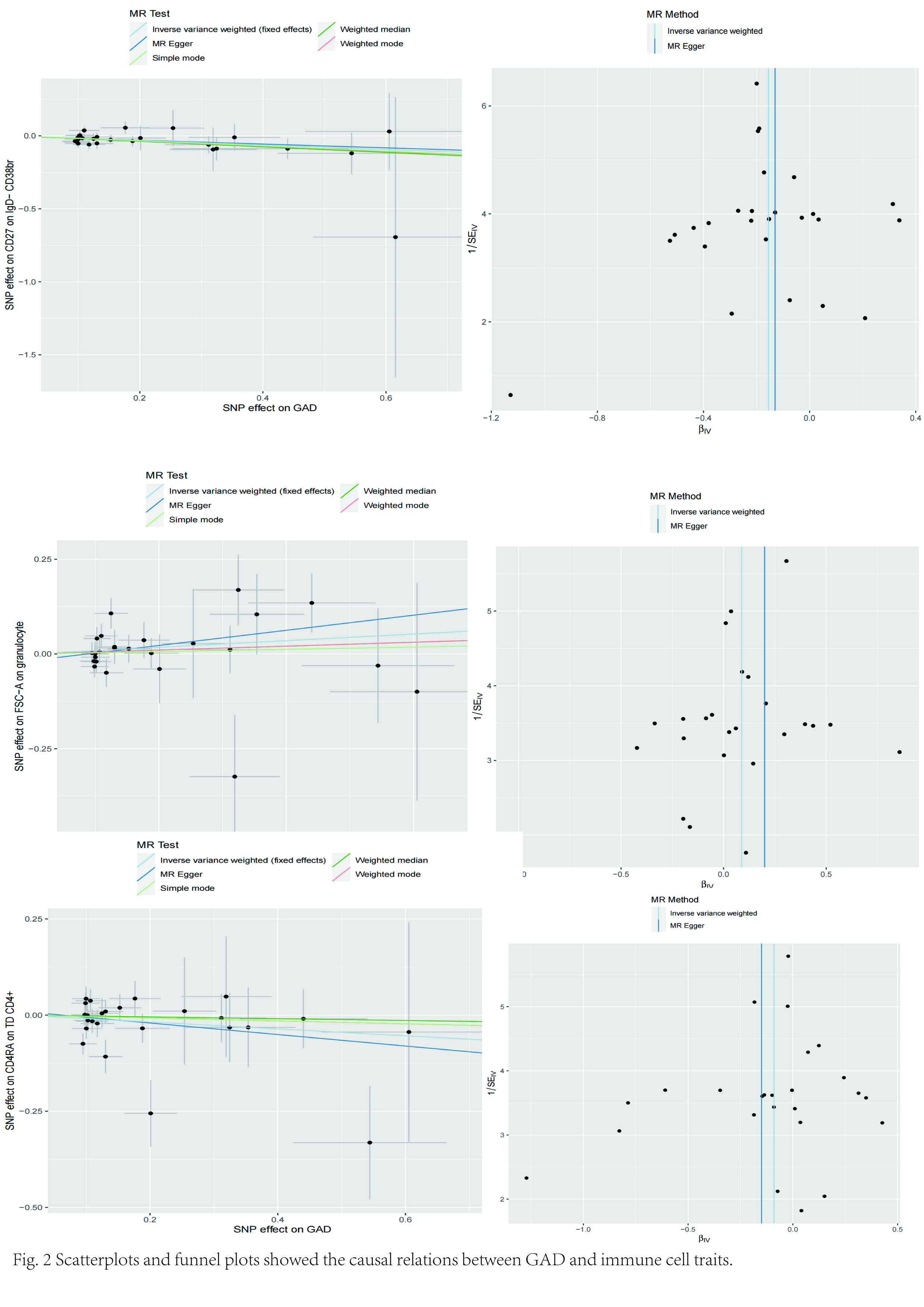

Supplement: Supplementary file 4 [file Image_4.tif]
